# A comprehensive expression profile of tRNA‐derived fragments in papillary thyroid cancer

**DOI:** 10.1002/jcla.23664

**Published:** 2020-12-17

**Authors:** Shiting Shan, Yuting Wang, Chunfu Zhu

**Affiliations:** ^1^ Nanjing Medical University Nanjing China; ^2^ Department of General Surgery The Affiliated Changzhou No.2 People's Hospital of Nanjing Medical University Changzhou China; ^3^ Dalian Medical University Dalian China

**Keywords:** expression profile, papillary thyroid cancer, tRNA‐derived fragments

## Abstract

**Background:**

The incidence of thyroid cancer has been on a rise. Papillary thyroid cancer (PTC) is the most common type of malignant thyroid tumor and accounts for approximately 85% of thyroid cancer cases. Although the genetic background of PTC has been studied extensively, relatively little is known about the role of small noncoding RNAs (sncRNAs) in PTC. tRNA‐derived fragments (tRFs) represent a newly discovered class of sncRNAs that exist in many species and play key roles in various biological processes.

**Methods:**

In this study, we used high‐throughput next‐generation sequencing technology to analyze the expression of tRFs in samples from PTC tissues and normal tissues. We selected four tRFs to perform qPCR to determine the expression levels of these molecules and make bioinformatic predictions.

**Results:**

We identified 53 unique tRFs and transfer RNA halves (tsRNAs). The 10 most upregulated tRFs and tsRNAs were tRF‐39‐I6D3887S1RMH5MI2, tRF‐21‐2E489B3RB, tRF‐18‐JMRPFQDY, tRF‐17‐202L2YF, tRF‐17‐VBY9PYJ, tRF‐18‐YRRHQFD2, tRF‐21‐WE884U1DD, tRF‐41‐EX2Z10I9BZBZOS4YB, tRF‐39‐HPDEXK7S1RNS9MI2, and tRF‐20‐1SS2P46I. The 10 most downregulated tRFs and tsRNAs were tRF‐31‐HQ9M739P8WQ0B, tRF‐43‐5YXENDBP1IUUK7VZV, tRF‐38‐RZYQHQ9M739P8WD8, tRF‐25‐9M739P8WQ0, tRF‐33‐V6Z3M8ZLSSXUD6, tRF‐27‐MY73H3RXPLM, tRF‐26‐DBNIB9I1KQ0, tRF‐38‐Z9HMI8W47W1R7HX, tRF‐40‐Z6V6Z3M8ZLSSXUOL, and tRF‐39‐YQHQ9M739P8WQ0EB. qPCR verification of cell lines and tissue samples yielded results consistent with the sequencing analysis. As tRF‐39 expression showed the maximum difference between PTC cells and normal cells, we chose this tRF to predict targets and perform functional tRF and tsRNA enrichment analysis.

**Conclusion:**

In this study, we provided a comprehensive catalog of tRFs involved in PTC and assessed the abnormal expression of these fragments. Through qPCR verification, tRF‐39‐0VL8K87SIRMM12E2 was found to be the most significantly upregulated tRF. Further tRF and enrichment analysis revealed that tRF‐39 was mostly enriched in the “metabolic pathways.” These preliminary findings can be used as the basis for further research studies based on the functional role of tRFs in patients with PTC.

## Graphical abstract

Background: The incidence of thyroid cancer has been on a rise. Papillary thyroid cancer (PTC) is the most common type of malignant thyroid tumour and accounts for approximately 85% of thyroid cancer cases. Although the genetic background of PTC has been studied extensively, relatively little is known about the role of small non‐coding RNAs (sncRNAs) in PTC. tRNA‐derived fragments (tRFs) represent a newly discovered class of sncRNAs that exist in many species and play key roles in various biological processes. Methods: In this study, we used high‐throughput next‐generation sequencing technology to analyse the expression of tRFs in samples from PTC tissues and normal tissues. We selected four tRFs to perform qPCR to determine the expression levels of these molecules and make bioinformatic predictions. Results: We identified 53 unique tRFs and transfer RNA halves (tsRNAs). The 10 most up‐regulated tRFs and tsRNAs were tRF‐39‐I6D3887S1RMH5MI2, tRF‐21‐2E489B3RB, tRF‐18‐JMRPFQDY, tRF‐17‐202L2YF, tRF‐17‐VBY9PYJ, tRF‐18‐YRRHQFD2, tRF‐21‐WE884U1DD, tRF‐41‐EX2Z10I9BZBZOS4YB, tRF‐39‐HPDEXK7S1RNS9MI2 and tRF‐20‐1SS2P46I. The 10 most down‐regulated tRFs and tsRNAs were tRF‐31‐HQ9M739P8WQ0B, tRF‐43‐5YXENDBP1IUUK7VZV, tRF‐38‐RZYQHQ9M739P8WD8, tRF‐25‐9M739P8WQ0, tRF‐33‐V6Z3M8ZLSSXUD6, tRF‐27‐MY73H3RXPLM, tRF‐26‐DBNIB9I1KQ0, tRF‐38‐Z9HMI8W47W1R7HX, tRF‐40‐Z6V6Z3M8ZLSSXUOL and tRF‐39‐YQHQ9M739P8WQ0EB. qPCR verification of cell lines and tissue samples yielded results consistent with the sequencing analysis. As tRF‐39 expression showed the maximum difference between PTC cells and normal cells, we chose this tRF to predict targets and perform functional tRF and tsRNA enrichment analysis. Conclusion: In this study, we provided a comprehensive catalog of tRFs involved in PTC and assessed the abnormal expression of these fragments. Through qPCR verification, tRF‐39‐0VL8K87SIRMM12E2 was found to be the most significantly upregulated tRF. Further tRF and enrichment analysis revealed that tRF‐39 was mostly enriched in the ‘metabolic pathways’. These preliminary findings can be used as the basis for further research studies based on the functional role of tRFs in patients with PTC.

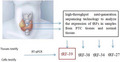

## 1 INTRODUCTION

Thyroid cancer is one of the most common malignant tumors in the endocrine system^1^ and has registered an annual growth rate of 2% in recent decades.^2^ Papillary thyroid cancer (PTC) is the most common type of malignant thyroid tumor, with more than 10 known subtypes that account for 85‐90% of all thyroid malignancies.^3^, ^4^ Fortunately, most PTCs are relatively inert and the 10‐year survival rate for patients undergoing a glandectomy or total thyroidectomy plus I131 treatment is greater than 95%.^5^ However, in approximately 10% of patients with PTC, recurrence or metastasis occurs and requires further intervention. Formulating treatments for PTC requires a thorough understanding of the molecular mechanisms underlying the pathogenesis and progression of the disease. Although the genetic background of PTC has been extensively studied, relatively little is known regarding the role of small noncoding RNAs (sncRNAs) other than microRNA (miRNA).^6^ An earlier study led to the discovery of a new class of sncRNA derived from tRNA, known as tRNA‐derived fragments (tRFs).^7^ tRFs have functions similar to those of miRNA, with the ability to directly bind to mRNA, inhibit protein translation, and cleave partially complementary targets.^8^ tRFs can also regulate gene expression via competitive binding with proteins of Ago (Argonaute) family, thereby affecting the silencing efficiency of target genes.^9^ In addition, tRFs and their analogs can affect the tumor gene mRNA binding protein Y‐box binding protein 1 (YBX1), by blocking its interaction with oncogenic mRNA and destabilizing oncogene mRNAs, thereby inhibiting tumor cell infiltration.^10^ tRFs can also regulate protein biosynthesis by affecting the translation. Furthermore, research studies have shown that tRFs can be used as guide RNAs in viral reverse transcription (RT) and can combine with primer RNA to initiate RT. When combined with cytochrome C, tRFs inhibit the formation of apoptotic bodies. Finally, studies have shown that tRFs exist in hematopoietic cells, lymphocytes, and the blood circulation system, suggesting that these fragments may play important role in the immune response.^11^, ^12^, ^13^

Previous studies have shown that tRFs play regulatory roles in various human diseases such as cancers, pathological stress injuries, metabolic diseases, viral infections, and diseases of nervous system.^14^, ^15^, ^16^ However, the understanding of how tRFs regulate cancer progression remains unknown.^17^ Previous studies have indicated that tRFs and transfer RNA (tRNA) halves are involved in cancers of breast, prostate, colorectum, liver, and pancreatic,^18^, ^19^, ^20^ although the potential role of tRFs and tRNA halves in thyroid cancer is yet to be elucidated. In this study, we sought to explore how the expression and function of tRFs regulate cancer cells in patients with PTC.

## 2 MATERIAL AND METHODS

### 2.1 Sample collection

Four patients (one woman, three men; age range, 30‐50 years), all of whom had undergone surgery for PTC at the Affiliated Changzhou No. 2 People's Hospital of Nanjing Medical University (Jiangsu, China), were recruited in 2019 to participate in this study (Table 1). All patients had no underlying disease and no history of previous surgical procedures. After the operation, they received hormone replacement therapy. Each patient provided written informed consent, and the ethics committee of Changzhou Second People's Hospital affiliated to Nanjing Medical University approved the study protocol. The specific stage of PTC in each patient was determined based on the thyroid cancer TNM staging system published by the American Joint Committee on Cancer in 2017.^21^ In total, four pairs of PTC specimens and adjacent control tissues (with a distance of 2 cm from the tumor) were collected for tRF sequencing and immediately stored in liquid nitrogen.

**TABLE 1:** Patient and tumor characteristics

Patient number	Age (years)	Sex	Tumor size (cm)	Tumor stage	Pathological description of tumor
1	45	Male	0.6	T1N0M0	Mini papillary carcinoma, no lymph node metastasis
2	37	Male	1.2	T1N1M0	Papillary carcinoma, left central lymph node 1/1 metastasis
3	48	Male	2.5	T2N0M0	Papillary carcinoma, no lymph node metastasis, tumor invasion of left recurrent laryngeal nerve
4	67	Female	0.9	T1N1M0	Papillary carcinoma, 1/4 lymph node metastasis in the central area, 1/1 lymph node metastasis in the left 2A area, and 6/19 lymph node metastasis in the left 4 area

Abbreviations: M, metastasis; N, node; T, tumor.

### 2.2 Types of tRNA‐derived small RNA (tsRNA)

**FIGURE 1:**
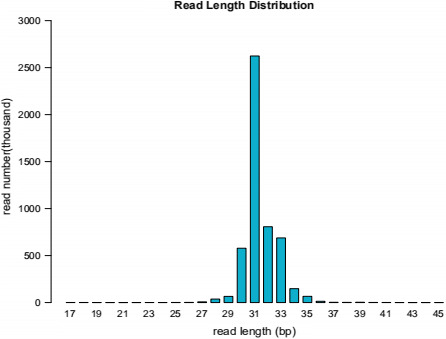
Statistics of known tsRNA length. The length distribution of mature tsRNA generated by tRNA shearing is shown in Figure 2. The length of tsRNA is mainly concentrated in the range of 16–35 nts

**FIGURE 2:**
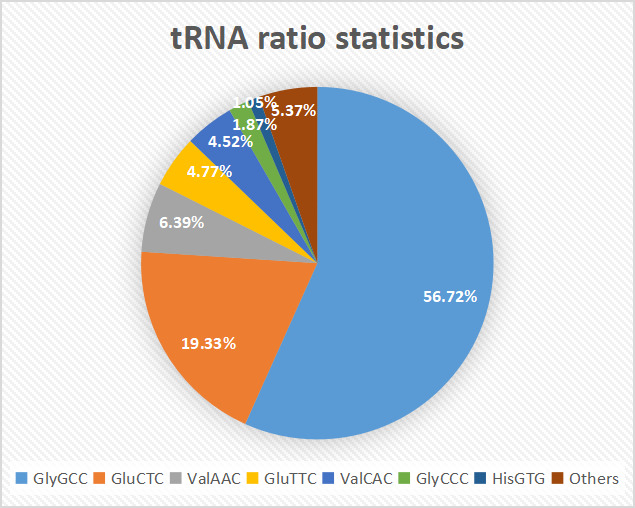
Statistics of the number of known tRNA. The known tRNA ratio distribution is shown in Figure 3: the light blue part represents the proportion of GlyGCC, the orange part represents GluCTC, the gray part represents ValAAC, the yellow part represents GluTTC, the blue part represents VAlCAC, the green part represents GlyCCC, dark blue The color part represents HisGTG, and the brown part represents other

**FIGURE 3:**
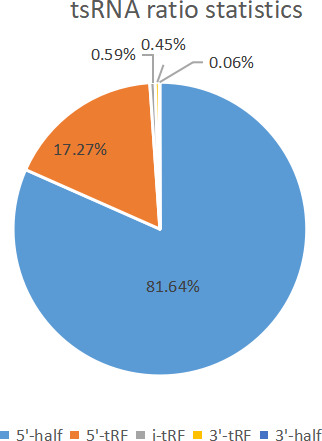
Statistics of the number of known tsRNA. The known tsRNA ratio distribution is shown in Figure 4: the light blue part represents the proportion of 5′‐half, the orange part represents 5′‐tRF, the gray part represents i'‐tRF, and the yellow part represents 3′‐tRF, the blue part represents 3′‐half

Small RNAs derived from tRNA (tsRNAs) refer to the nuclease, such as Dicer or Angiopoietin (ANG), specific to tRNAs in certain cells or tissues, or under certain conditions such as stress or hypoxia. tsRNA is a type of sncRNA, which are widely expressed in prokaryotic and eukaryotic transcriptomes and is produced by cleavage of mature or precursor tRNA at different sites. There are two main types of tsRNAs, tRFs, and tRNA halves.^22^ tsRNAs are divided into the following five categories based on their biogenesis and relative length: 1) 5′‐half: A 5′‐tRNA half‐molecule that is produced by ANG specifically cleaving at the anticodon loop of mature tRNA under various stress conditions; 2) 3′‐half: A 3′‐tRNA half‐molecule that is produced by ANG specifically cleaving at the anticodon loop of mature tRNA under various stress conditions; 3) 5′‐tRF: A fragment from mature tRNA in which the 5′ end is cleaved at the D‐loop or anticodon stem; 4) i‐tRF: a fragment originating from the inside of mature tRNA and t according to whether the 5′ sequence or the 3′ sequence contains anticodon cleavage sites, tsRNAs are divided into two subtypes, namely 5′ and 3′, with a length of 31‐40 nt. 5′ start at the 5′ end of the mature tRNA and end at the anticodon loop, whereas 3′ includes the anticodon loop and the 3′ end.^23^ The same type of tsRNA can be cut from multiple tRNAs, however, the function of such tsRNA derived from different tRNA sources will produce several types of tRFs with different biological functions. tRF can reduce the overall translation speed by 10% to 15%; part of the 5′ end from tRNAAla and tRNACys can be assembled into a G4 motif that can competitively bind eIF4G/eIF4A in the translation initiation complex, thereby tRF inhibit Cap‐dependent mRNA translation. The known tRNA and tsRNA ratio distributions are shown in Figures 1 and 2, respectively. As a sncRNA, the length of mature tsRNA generated by tRNA shearing generally ranges from 16 to 35 nt (Figure 3).

### 2.3 tRF sequencing

As tRFs are short fragments, the library was constructed using the following protocol: the total RNA or purified tsRNA fragments were extracted from samples with the 3′ end and the 5′ connector joined successively. Then, complementary DNA (cDNA) was synthesized using RT, followed by PCR expansion. The target fragment library was recovered via gel extraction and sequenced on the computer. Illumina HiSeq 2500 was used to sequence the raw data reads. The joints at both ends of the reads were removed and low‐quality reads and reads with a fragment length < 15 nt were also removed to complete the initial data filtering and to obtain high‐quality data (clean reads). Clean reads were compared with the reference genome to obtain a genome‐wide read distribution map, and were annotated according to sncRNA classification. The expressions of identified tsRNAs and cluster tsRNAs were calculated, and tsRNAs were analyzed for differential expression between samples.

### 2.4 Quantitative RT‐PCR (qRT‐PCR)

We selected four tRFs for PCR verification. The sequences of the four tRFs are shown in Table 2. First, we extracted total RNA from PTC cell lines, PTC tissue, normal thyroid cell lines, and normal tissue using RL lysate. The RNA was reverse‐transcribed at 42°C for 60 min followed by at 70°C for 10 min, using the Bulge‐Loop miRNA qRT‐PCR Starter Kit (Guangzhou Ribobio Biological Technology, China). Next, qPCR was performed using U6 small nuclear RNA (snRNA) as an internal control. After adding the cDNA and forward and reverse primers designed specifically for each tRF by Ribobio, the fragments were incubated at 95°C for 10 min, followed by 60 cycles of amplification at 95°C for 2 s, 60°C for 20 s, and 70°C for 10 s on the Roche LightCycler 480 Real‐Time PCR System (Roche LightCycler 480 system, Basel, Switzerland). Finally, the relative expression levels of each specific tRF in each sample group were normalized using the 2^−ΔΔCt^ method.

**TABLE 2:** tRFs and tiRNAs selected for validation via quantitative PCR

tsRNA_ID	Sequence
tRF‐27‐PIR8YP9LON3	GCACTGGTGGTTCAGTGGTAGAATTCT
tRF‐34‐YSV4V47Q2WW1J1	TTCCGTAGTGTAGTGGTTATCACGTTCGCCTCAC
tRF‐38‐0VL8K87SIRMM12V	ACCGCCGCGGCCCGGGTTCGATTCCCGGTCAGGGAACC
tRF‐39‐0VL8K87SIRMM12E2	ACCGCCGCGGCCCGGGTTCGATTCCCGGTCAGGGAACCA

Abbreviations: tRF, transfer RNA‐derived fragment; tsRNA, tRNA‐derived small RNA.

### 2.5 Predicted targets of tRF and tsRNA

tsRNA target gene prediction mainly considers the complementary pairing of tsRNA and mRNA 3′ untranslated regions. Different software programs predict different target genes based on the complementary pairing of tsRNA and its target site. Therefore, we used a combination of the following three unique software programs to predict targets: MiRanda (http://www.microrna.org/microrna/home.do), RNAhybrid (http://www.bibiserv.cebitec.unibielefeld.de/rnahybrid/), and targetScan (http://www.targetscan.org/). We then screened and sorted the results from the three programs and used the prediction results as the candidate target genes of tsRNA.

### 2.6 Functional tRF and tsRNA enrichment analysis

Biological pathway analysis was performed in conjunction with the Kyoto encyclopedia of genes and genomes (KEGG) Biological Pathway Database (https://www.kegg.jp/). We placed the candidate target genes into the biological pathway for comprehensive analysis and analyzed the degree of influence and regularity of the functional variation on the biological pathway. The gene function enrichment analysis process was thus, divided into two steps, a KEGG biological pathway annotation, and an enrichment analysis. For this analysis, *p* values were calculated using Fisher's exact test, with a *p* value of 0.05 used as the significance threshold. Significant signal transduction and disease pathways were identified, thereby providing distribution information and significance status for gene sets in the KEGG category. Gene ontology analysis was used to perform functional annotation for each gene and calculate the most significant function in a specific series of genes via statistical analysis, for example, by using hypergeometric distribution.

## 3 RESULTS

### 3.1 tRF sequencing

**FIGURE 4:**
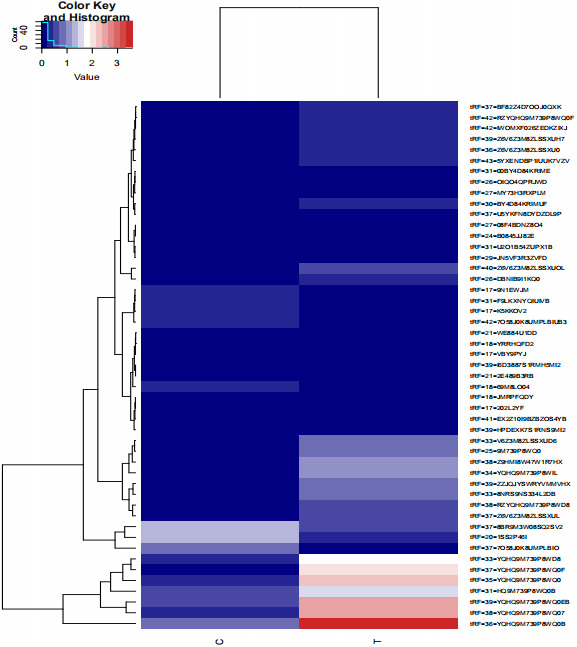
tsRNA heat map of different expression levels between samples. Heat map indicating the expression levels of various tRFs and tiRNAs.Brick red represents high expression of tsRNA in the sample, navy blue represents low expression. Each square represents a gene, and its color represents the amount of expression of the gene. The higher the amount of expression, the darker the color

We detected thousands of differentially expressed tRFs in PTC through high‐throughput next‐generation sequencing technology. Here, we present 53 differentially expressed tRFs (Table 3), including 19 downregulated tRFs and tsRNAs, and 34 upregulated tRF and tsRNAs, which exhibited abundant expression and were significantly different between the thyroid cancer samples and normal tissue samples. These 53 tRFs were derived from different types of tRNAs and belonged to different tRF types. A heat map representing the expression in various samples is shown in Figure 4.

**TABLE 3:** The 53 tRFs and tiRNAs expressed within thyroid cancer samples

tsRNA_ID	tRNA	tsRNA type	Fold change, T/N	*p* value
Upregulated tsRNA				
tRF‐39‐I6D3887S1RMH5MI2	ArgTCT	3′‐tRF	3.9069	0.014928
tRF‐21‐2E489B3RB	MetCAT	3′‐half	3.8826	0.021853
tRF‐18‐JMRPFQDY	LysCTT	i‐tRF	3.6147	0.014696
tRF‐17‐202L2YF	PheGAA	i‐tRF	3.5546	0.037522
tRF‐17‐VBY9PYJ	GlyGCC	i‐tRF	3.5025	0.022305
tRF‐18‐YRRHQFD2	GlyCCC	3′‐tRF	3.4594	0.037242
tRF‐21‐WE884U1DD	TyrGTA	3′‐tRF	3.4594	0.039407
tRF‐41‐EX2Z10I9BZBZOS4YB	ValTAC	i‐tRF	3.4263	0.041616
tRF‐39‐HPDEXK7S1RNS9MI2	ArgCCG	3′‐tRF	3.1699	0.042129
tRF‐20‐1SS2P46I	MetCAT	3′‐half	2.8836	0.012036
tRF‐17‐9N1EWJM	GluCTC	i‐tRF	2.3410	0.016507
tRF‐37‐7O58J0K8UMPLBIO	LeuTAA	5′‐half	2.3172	0.018091
tRF‐31‐F9LKXNYQIUIVB	CysGCA	5′‐tRF	2.2257	0.033673
tRF‐42‐7O58J0K8UMPLBIUB3	LeuTAA	5′‐tRF	2.1699	0.039598
tRF‐17‐K5KKOV2	AsnGTT	3′‐tRF	2.0875	0.015291
tRF‐18‐69M8LO04	ArgTCT	5′‐tRF	2.0395	0.047687
tRF‐37‐8BR9M3W08SQ2SV2	ArgTCT	3′‐tRF	1.5567	0.047326
tRF‐38‐0VL8K87SIRMM12V	GluTTC	3′‐tRF	2.3693	0.039325
tRF‐39‐0VL8K87SIRMM12E2	GluTTC	3′‐tRF	3.2978	0.040484
Downregulated tsRNA				
tRF‐31‐HQ9M739P8WQ0B	GluTTC	i‐tRF	−2.0213	0.040330
tRF‐43‐5YXENDBP1IUUK7VZV	HisGTG	i‐tRF	−2.2395	0.040484
tRF‐38‐RZYQHQ9M739P8WD8	GluTTC	i‐tRF	−2.2630	0.033734
tRF‐25‐9M739P8WQ0	GluTTC	i‐tRF	−2.3933	0.030280
tRF‐33‐V6Z3M8ZLSSXUD6	GluTTC	i‐tRF	−2.4111	0.029211
tRF‐27‐MY73H3RXPLM	GlyTCC	i‐tRF	−2.4374	0.044427
tRF‐26‐DBNIB9I1KQ0	SerGCT	i‐tRF	−2.4703	0.045635
tRF‐38‐Z9HMI8W47W1R7HX	GluTTC	i‐tRF	−2.6933	0.022304
tRF‐40‐Z6V6Z3M8ZLSSXUOL	GluTTC	i‐tRF	−2.8908	0.019767
tRF‐39‐YQHQ9M739P8WQ0EB	GluTTC	i‐tRF	−3.0422	0.032495
tRF‐29‐JN5VF3R3ZVFD	AlaTGC	i‐tRF	−3.1699	0.041342
tRF‐33‐8NRS9NS334L2DB	GluTTC	i‐tRF	−3.1802	0.039608
tRF‐34‐YSV4V47Q2WW1J1	ValCAC	i‐tRF	−3.2677	0.032495
tRF‐37‐Z6V6Z3M8ZLSSXUL	GluTTC	i‐tRF	−3.2750	0.013965
tRF‐26‐OIQO4QPRJWD	SerGCT	i‐tRF	−3.3219	0.037197
tRF‐27‐PIR8YP9LON3	ValCAC	5′‐tRF	−3.3563	0.010854
tRF‐31‐U2O1B54ZUPX1B	LysTTT	3′‐tRF	−3.3923	0.032766
tRF‐31‐00BY4D84KRIME	SerGCT	i‐tRF	−3.4594	0.032259
tRF‐24‐B0845JJ82E	SerTGA	i‐tRF	−3.5546	0.043139
tRF‐27‐08F4BDNZ8O4	ThrTGT	i‐tRF	−3.6147	0.044530
tRF‐33‐YQHQ9M739P8WD8	GluTTC	i‐tRF	−3.6358	0.020345
tRF‐38‐YQHQ9M739P8WQ07	GluTTC	i‐tRF	−3.6894	0.007831
tRF‐36‐YQHQ9M739P8WQ0B	GluTTC	i‐tRF	−3.7654	0.018644
tRF‐42‐RZYQHQ9M739P8WQ0F	GluTTC	i‐tRF	−3.8074	0.025013
tRF‐37‐BF82Z4D7OOJ0QXK	HisGTG	i‐tRF	−3.8074	0.034026
tRF‐42‐IWOMXF026ZEDKZIXJ	HisGTG	i‐tRF	−3.8244	0.042981
tRF‐35‐YQHQ9M739P8WQ0	GluTTC	i‐tRF	−4.0356	0.010854
tRF‐36‐Z6V6Z3M8ZLSSXU0	GluTTC	i‐tRF	−4.0589	0.046194
tRF‐39‐ZZJQJYSWRYVMMVHX	GluTTC	i‐tRF	−4.1015	0.004510
tRF‐34‐YQHQ9M739P8WIL	GluTTC	i‐tRF	−4.1224	0.006380
tRF‐37‐U5YKFN8DYDZDL9P	ValTAC	i‐tRF	−4.1293	0.032939
tRF‐30‐BY4D84KRIMUF	SerGCT	i‐tRF	−4.2854	0.009510
tRF‐39‐Z6V6Z3M8ZLSSXUH7	GluTTC	i‐tRF	−4.4594	0.039325
tRF‐37‐YQHQ9M739P8WQ0F	GluTTC	i‐tRF	−4.5495	0.011287

Abbreviations: i‐tRF, tRNA half; T/N, tumor/normal tissue; tiRNA, transfer RNA half; tRF, transfer RNA‐derived fragment; tsRNA, tRNA‐derived small RNA.

### 3.2 Quantitative RT‐PCR (qRT‐PCR)

**FIGURE 5:**
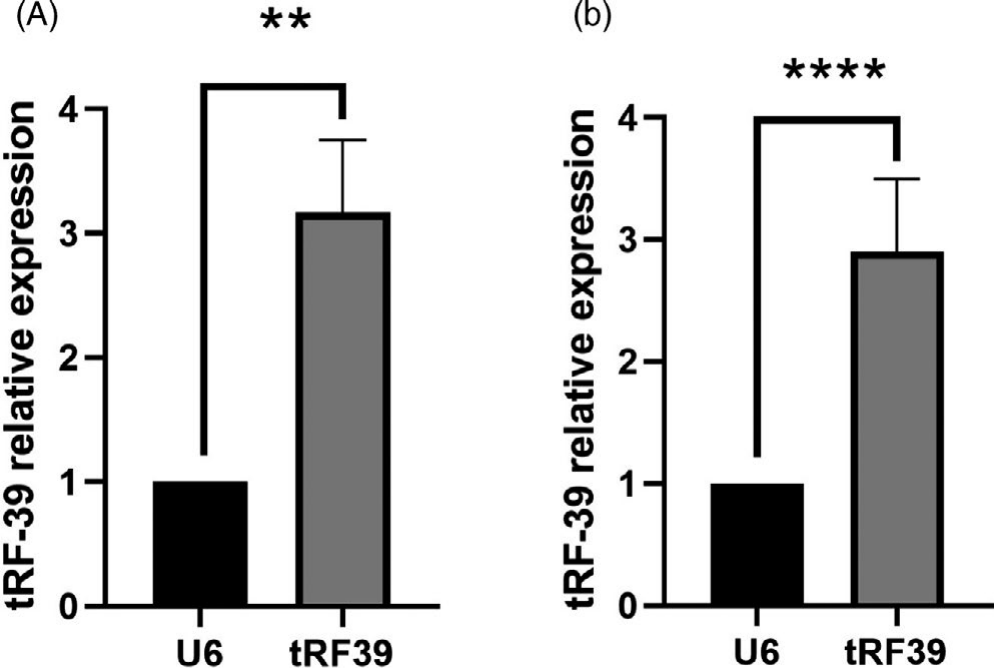
The expression of tRF‐39. tRF‐39 was upregulated in PTC cell lines and tissue samples. (A) tRF‐39 was upregulated in PTC cells, **p < 0.01; (B) tRF‐39 was upregulated in PTC tissue samples, ****p < 0.0001. The data are expressed as mean ± SD

For further qPCR verification, we chose four tRFs (tRF‐38‐0VL8K87SIRMM12V, tRF‐39‐0VL8K87SIRMM12E2, tRF‐27‐PIR8YP9LON3, and tRF‐34‐YSV4V47Q2WW1J1), which were expressed abundantly and were significantly different between the thyroid cancer samples and normal tissue samples. qPCR analysis demonstrated that tRF‐38‐0VL8K87SIRMM12V and tRF‐39‐0VL8K87SIRMM12E2 were upregulated whereas tRF‐34‐YSV4V47Q2WW1J1 and tRF‐27‐PIR8YP9LON3 were downregulated in the thyroid cancer cell lines. Also, we perform qPCR in 10 pair tissue samples whom had undergone surgery for PTC at the Affiliated Changzhou No. 2 People's Hospital of Nanjing Medical University. As a result, tRF‐38‐0VL8K87SIRMM12V and tRF‐39‐0VL8K87SIRMM12E2 were upregulated and tRF‐34‐YSV4V47Q2WW1J1 and tRF‐27‐PIR8YP9LON3 were downregulated in PTC tissues sample, which was consistent with the cell line analysis. tRF‐39 expression showed the maximum difference in PTC cell lines and PTC tissue samples. The results of qPCR are shown in Figure 5.

### 3.3 Predicted targets of functional tRF and enrichment analysis

**FIGURE 6:**
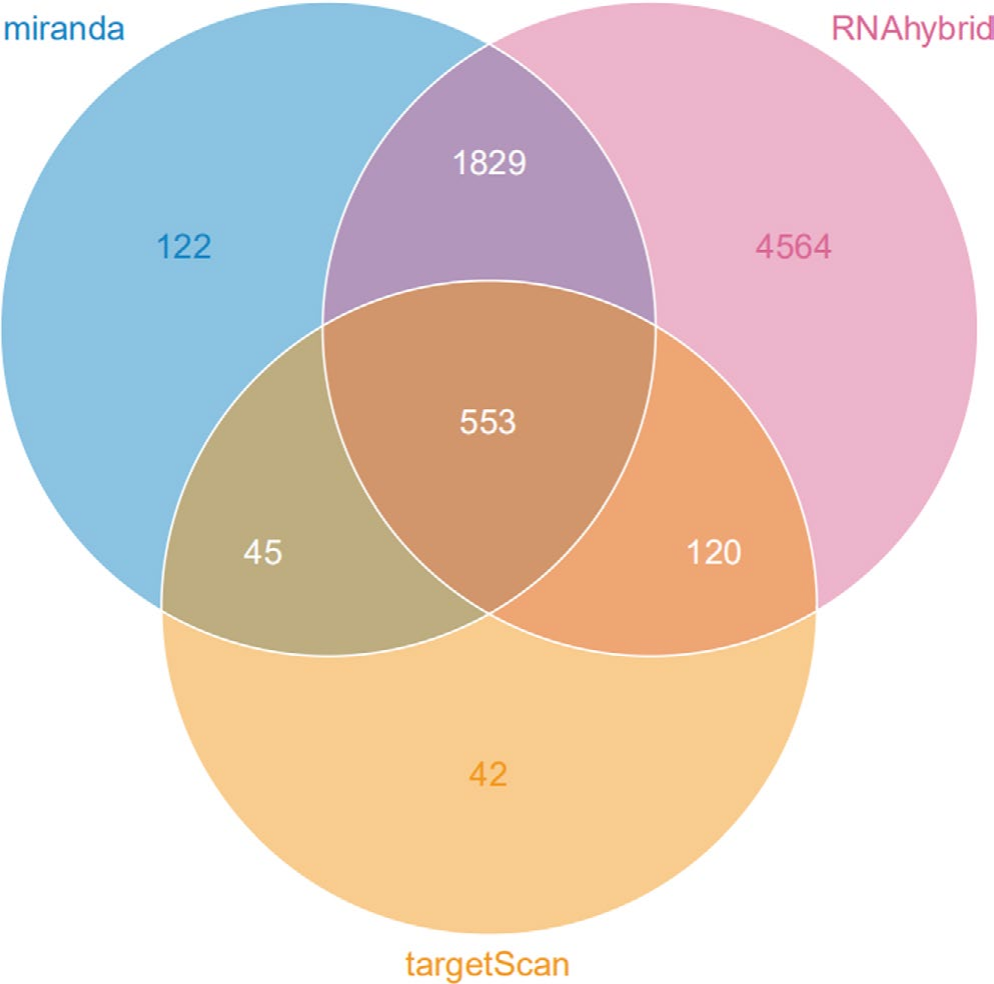
Target Gene Prediction Venn Diagram. The intersection of the target genes of tsRNA shown in the Venn diagram (venn/Venn diagram) in the three softwares

**FIGURE 7:**
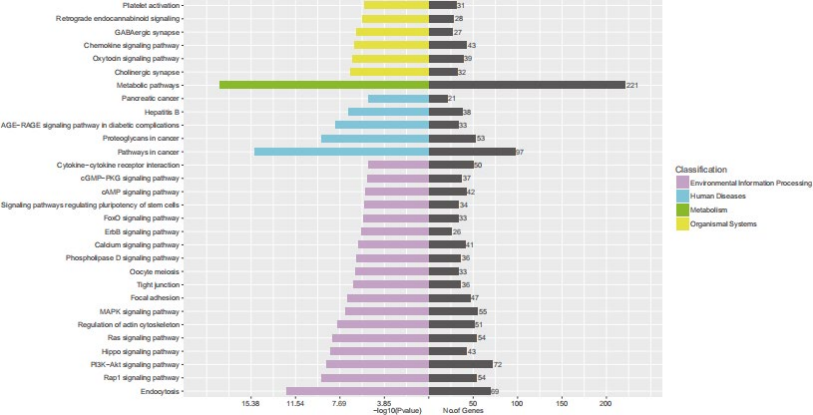
Enrichment diagram of KEGG pathway between samples. In the figure, the purple part represents Environmengtal information processing, the blue part represents Human disease, the green part represents Metabolism, and the yellow part represents Organicismal Systems

**FIGURE 8:**
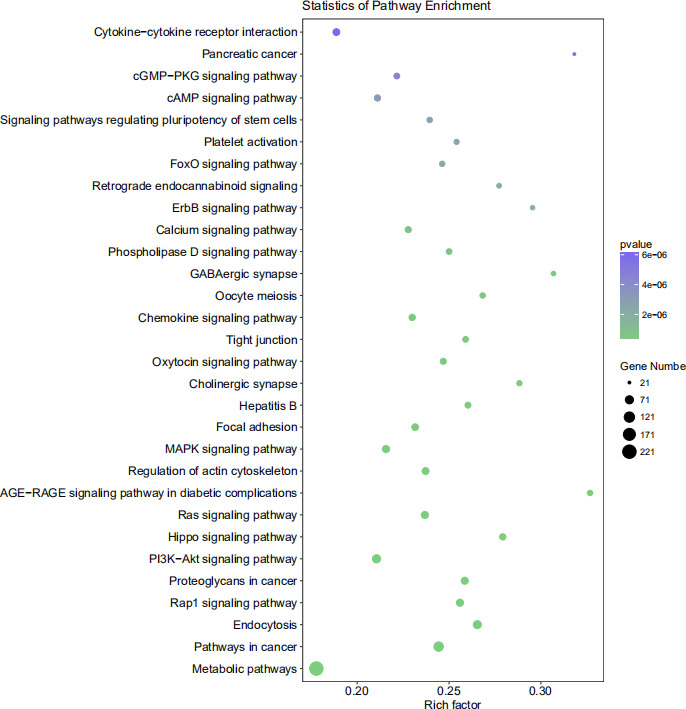
Candidate target gene KEGG pathway bubble chart. In the figure, the abscissa indicates the ratio of enriched differential genes to the background genes of the pathway, and the ordinate indicates the pathway name; the size of the dots in the graph indicates the number of enriched differential genes, and the color indicates the *p* value

The target genes of tsRNAs that were produced from all three software programs and were used in the study can be seen in Figure 6. For target gene prediction, tRF‐39‐0VL8K87SIRMM12E2 was predicted to have 553 target genes by miRanda, RNAhybrid, and TargetScan. KEGG analysis was also performed on the target genes and an example of the KEGG channel analysis results is shown in Figure 7. tRF‐39‐0VL8K87SIRMM12E2 was mostly enriched in the “metabolic pathways” section of the figure. Lastly, a candidate target gene KEGG pathway bubble chart showing the proportion of enriched differential genes in the background genes of the pathway is presented in Figure 8.

## 4 DISCUSSION

As sequencing technology has improved, research into sncRNAs has progressed and led to the discovery of tRFs.^24^ Since the initial discovery of these fragments, tRFs have been described in many species and organisms, as well as in cell lines including various cancer cell lines.^25^ More specifically, studies have shown that some tRFs may play a role in cancer suppression by combining with the mRNA binding protein YBX1, which is overexpressed in many cancer types and has been implicated in many key cellular pathways.^26^ In colorectal cancer for example, the combination of tRFs and miR‐1280 suppresses cancer growth and metastasis by repressing Notch signaling pathways that support cancel stem cell phenotypes.^27^ Other studies have demonstrated that tRFs are expressed in patients with prostate cancer, with survival curve analyses showing that tRF expression affects both progression‐free survival and prognosis.^28^

Increasing number of studies have demonstrated the important role played by tRFs tumors. Some tRFs promote cell proliferation and cell cycle progression by regulating the expression of oncogenes and other tRFs, thereby inhibiting cancer progression. Mechanistic studies have shown that tRFs can bind to RNA binding proteins, such as the YBX1, and prevent transcription, inactivate the initiation factor eIF4G/A, promote ribosomal protein translation, and activate aurora kinase A, a modulator of mitosis. Therefore, tRFs and tsRNAs regulate the occurrence and development of cancers of lungs, colorectum, prostate, breast, ovaries, and B cells, as well as chronic lymphocytic leukemia, among others.^29^ In breast cancer, tsRNA‐19, tsRNA‐29, tsRNA‐46, and tsRNA‐112 selectively respond to the expression of RUNX1 tumor suppressor. We found that ts‐112 and RUNX1 are correlated in normal breast epithelium and breast cancer cell lines, which is consistent with the tumor‐related activity of ts‐112 and the tumor suppressor activity of RUNX1. The inhibitory effect of ts‐112 in MCF10CA1a (an invasive breast cancer cell line) reduced proliferation significantly, while the ectopic expression of ts‐112 in MCF10A (normal breast epithelial cells) increased proliferation significantly. These results verified the carcinogenic potential of ts‐112. In addition, RUNX1 may inhibit ts‐112 to prevent hyperproliferation of breast cancer epithelial cells, thereby confirming its role in maintaining the breast epithelium.^30^ In gastric cancer, ‐5034‐GluTTC‐2 is downregulated in gastric cancer tissue and plasma, and its level is significantly related to tumor size. The overall survival rate of patients with low ‐5034‐GluTTC‐2 expression is significantly lower than that of patients with higher expression.^31^ These results indicate that ‐5034‐GluTTC‐2 has an important relationship with the occurrence and development of gastric cancer. Studies have shown that the dysregulation of tsRNA expression is a concomitant event in the development of disease, and mature tRFs may play carcinogenic and tumor‐suppressing roles in chronic lymphocytic leukemia.^32^

In this study, we explored the composition and expression of tRFs in patients with PTC. We screened 53 tRFs and tsRNAs from thyroid cancer samples and normal tissue samples and found 19 downregulated tRFs and tsRNAs, and 34 upregulated tRFs and tsRNAs. Through qPCR verification, we confirmed that tRF‐39‐0VL8K87SIRMM12E2 and tRF‐38‐0VL8K87SIRMM12V are upregulated in PTC, whereas and tRF‐34‐YSV4V47Q2WW1J1 and tRF‐27‐PIR8YP9LON3 are downregulated, which is consistent with our sequencing data. Repeated qPCR verification confirmed the largest expression difference of tRF‐39‐0VL8K87SIRMM12E2 between thyroid cancer cells and normal cells. In addition, we used the software programs MiRanda, RNAhybrid, and targetScan to predict the targets of differentially expressed tRFs and tsRNAs. Our analysis confirmed that upregulated tRF‐39‐0VL8K87SIRMM12E2 was mainly involved in metabolic pathways and in cancer‐related signaling pathways. Previous studies have focused on effect of tRFs on tumor occurrence and development, but little is known regarding how tRFs affect the metabolic pathways. Based on the results of our study, further research is warranted to determine how tRFs affect the occurrence and development of PTC by affecting the biological metabolism. In the near future, we plan to conduct such studies to confirm and expand the findings of the present study. This study had several limitations. The results are based only on tRF sequencing analysis and bioinformatics predictions and require validation based on in vitro and in vivo models. Additionally, the small sample size of this study led to large statistical differences between samples, necessitating the need for further studies based on large clinical cohorts.

## 5 CONCLUSION

In this study, we provided a comprehensive expression profile of tRFs in PTC and identified several potential avenues of further research, including the biological role and marker potential of tRFs in thyroid cancer.

## CONFLICT OF INTEREST

The authors indicated no potential conflicts of interest.

## Data Availability

All data generated or analyzed during this study are included in this published article [and its supplementary information files].
